# Demographic stratification of Type 2 diabetes and comorbidities in district healthcare in KwaZulu-Natal

**DOI:** 10.4102/safp.v63i1.5218

**Published:** 2021-04-20

**Authors:** Lauren Chetty, Nalini Govender, Ganesan M. Govender, Poovendhree Reddy

**Affiliations:** 1 Department of Community Health Studies, Faculty of Health Sciences, Durban University of Technology, Durban, South Africa; 2 Department of Basic Medical Sciences, Faculty of Health Sciences, Durban University of Technology, Durban, South Africa; 3 General Outpatients Department, R.K. Khan Hospital, Durban, South Africa

**Keywords:** type 2 diabetes mellitus, demographics, comorbidities, prevalence, healthcare, non-communicable diseases, epidemiology, disease burden

## Abstract

**Background:**

Diabetes has been reported as the second leading cause of death and the top leading cause of death amongst women in South Africa; it is important to evaluate any epidemiological or demographic transition related to diabetes. This study evaluated the demographically stratified prevalence of type 2 diabetes mellitus (T2DM) and existing comorbidities amongst an outpatient population in a district healthcare facility in Kwazulu-Natal (KZN).

**Methods:**

This retrospective cross-sectional study was conducted at a district hospital, and a retrospective record review of all outpatients who reported to the hospital to be treated for T2DM between the period, August 2018–January 2019, was used. Data, such as age, sex, ethnicity and any coexisting morbidity, were collected from outpatient hospital registers and electronically captured using a record review tool.

**Results:**

There were significantly more female patients (3072) compared to male patients (1050) (*p* < 0.001) with a mean age of 59.21 years. Hypertension (77.9%) and cardiovascular problems (11.16%) were most frequent. Approximately 84% of women presented with T2DM and either one or two morbidities simultaneously. Female patients were at significantly higher risk of presenting with hypertension (odds ratio [OR] = 1.44, 95% confidence interval [CI]: 1.20;1.71), whilst their risk for cardiovascular problems was significantly lower compared to male patients (OR = 0.67, 95% CI: 0.54;0.83).

**Conclusion:**

The prevalence of T2DM and comorbidities differed by demographic factors, such as sex, ethnicity and age. There is a need for flexible and adaptive approaches for the prevention and management of T2DM cases in order to allocate medical resources efficiently and according to the true burden of disease because of T2DM complications.

## Background

The International Diabetes Federation (IDF) estimates that 693 million adults will be living with diabetes mellitus (DM) by 2045.^1^ It is predicted that by 2030, developing countries will experience as much as a 69% increase in new diagnoses.^2^ South Africa (SA) is particularly vulnerable to this epidemic given the increase in urbanisation, prevalence of obesity and physical inactivity and the strained healthcare system already burdened by communicable diseases, such as human immunodeficiency virus (HIV) and tuberculosis (TB).^3^ It is imperative to continue tracking both the prevalence and incidence of type 2 diabetes mellitus (T2DM), given the impact on healthcare resources and the implications for the emerging SA National Health Insurance (NHI) policy. Type 2 diabetes mellitus was reported to be the fifth leading cause of death in 2012–2013 (4.4% – 4.8%) and was ranked as the second leading cause of death for women.^4^ Barely 2 years later (2015–2016), it is the overall second leading cause of death (5.4% – 5.6%) and ranked as the top leading cause of death amongst women (7.2%).^4^ District Health Information System (DHIS) data showed that the provincial crude prevalence of T2DM in KwaZulu-Natal (KZN) was 12.5% which increased to 34.1% when patients with private medical aid and undiagnosed T2DM were considered.^5^ Risk factors linked to T2DM are extensively reported.^6,7^ However, it is important to evaluate any demographic transition related to T2DM, particularly for mitigation and management.

In SA, there was a 5.5% increase in the prevalence of T2DM amongst people aged 30 years and older between 2000 and 2012.^8^ Likewise, in Africa, the prevalence is increased amongst people aged between 40 and 60 years in contrast to those older than 60 years.^9^ Sub-Saharan African (SSA) studies demonstrated a peak prevalence in the oldest age group (> 65 years),^10,11,12^ in contrast to others who showed a peak prevalence in the 45–64 years age group.^13,14,15^ From global estimates, it is likely that the male excess previously reported for SSA is likely to increase by 2025.^16^ However, it is evident from available data that the gender distribution varies between and within populations with no obvious trend.^17^ Ethnicity and genetic history have been reported as a significant risk factor in T2DM.^18^ Asians are reported to have an increased susceptibility for T2DM,^19^ which may be similar for SA Indians. Moreover, this T2DM incidence may be increasing amongst Africans as a result of increasing urbanisation and socio-economic conditions coupled with poor nutritional choices.^9^ An evaluation of demographic data associated with the incidence and prevalence of T2DM is essential in advancing the precision in the prediction of incidence, complications, mortality and frequency of atypical variants.^20^

Previous studies suggest that chronic complications presented with T2DM reduce the quality of life, increase diabetes-related mortality and pose a significant healthcare burden.^21,22^ The spectrum of various comorbidities linked with T2DM requires correct management and is critical in African countries, including South Africa, where limited healthcare resources require strategic allocation. T2DM-related comorbidities include micro- or macro-vascular complications, such as cardiovascular diseases, blindness, peripheral neuropathy and kidney disease,^23^ which subsequently increases the risk of strokes, heart attacks and amputations.^24^ Other conditions include adverse oral health, arthritis, vision-related issues, depression, slow wound healing, fatigue and hypoglycaemic episodes.^25^ Diabetics may present with several of the above conditions simultaneously, contributing to increased mortality and morbidity.^23,26^ In addition, demographic factors, such as age, sex and ethnicity, should be considered in relation to comorbidities, given that South Africa is facing an increased burden of non-communicable diseases.^27^ KwaZulu-Natal has the largest population of Indians and the highest reported HIV and TB prevalence in SA,^5^ predisposing them to increased susceptibility. This study included a retrospective assessment of patients who attended a regional hospital in the EThekwini district (KZN, SA), with the aim of determining the demographic prevalence of T2DM and existing comorbidities.

## Methods and materials

### Study design and site

This retrospective study was conducted at a district hospital located in the eThekwini health district. The hospital has a catchment population of over 1 500 000 people who are amongst the poorest in the eThekwini district. The hospital has approximately 36 000 admissions in a year, and 600 000 outpatients are treated annually.^28^ It serves a population of approximately 240 000.

### Study population and sampling strategy

All outpatients who were treated for T2DM between the period August 2018 and January 2019 were included in the study. Permission was sought from the Department of Health and hospital management to conduct the study. Once permission was received, a retrospective review on hospital registers for the 6-month period was carried out to determine the prevalence of T2DM. Data, such as age, gender and ethnicity, were collected and electronically captured using a data capturing tool.

### Inclusion criteria

All participants who were:

- treated with T2DM within the 6 months (Aug 2018 – Jan 2019) under study
- reported to R.K. Khan Hospital for chronic treatment for T2DM.

### Exclusion criteria

- Type 1 diabetes
- Gestational diabetes

### Data collection

A retrospective record review of all outpatients who attended the DM clinic in the hospital for chronic treatment between the period August 2018 and January 2019 was conducted. Data, such as age, sex, ethnicity and any coexisting morbidity, were collected from outpatient hospital registers (paper-based) for a period of 6 months and electronically captured using a record review tool. Data were limited by the information available in the hospital register; demographic variables such as gender, age and ethnicity were available. Clinical data included a record of specific comorbidities such as hypertension, cardiac problems, epilepsy, asthma, arthritis, anaemia and mental health. Cardiovascular problems included congenital heart disease, coronary artery disease, heart failure, heart attack, heart valve disease, cardiomyopathy, atherosclerosis and ischemic heart disease. However, clinical data for other related T2DM-associated morbidities (e.g. oral, ocular and foot ulcers) were not available from the outpatient registers.

### Data analysis

Data were captured using Microsoft Excel using double entry procedures, cleaned through range checking and spot checking and coded for data analysis. Data were analysed using STATA version 12 (Statacorp). Descriptive statistics included frequency counts, percentages, mean and standard deviation. T2DM cases from the hospital register were stratified by ethnicity, age and gender to determine the demographic profile. A test of proportions was used to evaluate differences between demographic variables and sex. Patients with early onset DM were defined as those patients who were first diagnosed with DM before 45 years old. Age was dichotomised by sex, ethnicity and pre-existing comorbidities as both a continuous and categorical variable. The *t*-test was used for comparison of age stratified by sex and pre-existing comorbidities, whilst the chi-squared test of proportions was used to compare T2DM stratified by sex, ethnicity, age and comorbidities. A variable was created to represent the total number of pre-existing comorbidities for each patient as reflected in the hospital register. Multivariate logistic regression models, adjusted for age, were run using the comorbidity as the dependent variable and gender and ethnicity as the independent variables. In addition, we used the frequency of pre-existing comorbidities as a dependent variable with sex and ethnicity as the independent variables. A 95% confidence interval (CI) was reported and *p* ≤ 0.05 was considered statistically significant.

### Ethical considerations

The study was approved by the Durban University of Technology Institutional Research Ethics Committee (IREC) (REC 112/19) and the KwaZulu-Natal Department of Health.

## Results

A total of 4122 (*N*) outpatients were presented with T2 DM between August 2018 and January 2019 at the district hospital. There were significantly more female patients (3072) compared to male patients (1050) (*p* < 0.001) with a mean age of 59.21 years (Table 1). Overall, approximately 10% of patients were presented with early onset DM (< 45 years old), affecting significantly more women than men (309 vs. 132). Most of the new cases presenting with T2DM over the 6-month period were between 55 years and 64 years old (31.59%) with 77.42% of all outpatients between the ages of 45 years and 74 years (Table 1). Diabetic outpatients seeking treatment at the hospital dropped from 644 in August 2018 to 584 in September 2018. A marked increase was noted between October (834) and November (1222), with fewer patients in December (408) and January (429). This reduction may be related to the poor access during the holiday season.

**TABLE 1:** Age profile of type 2 diabetes mellitus patients presenting at the district hospital stratified by sex.

Demographic	Total		Male		Female		*p* *
	*n*	%	*n*	%	*n*	%	
Total	4122	100.00	1050	25.47	3072	74.53	< 0.005
Age (mean, SD)	59.21	12.84	57.92	12.68	59.66	12.86	0.245
Early onset DM†	441	10.70	132	12.57	309	10.06	0.012
**Age category (years)**							
0–15	15	0.36	4	0.38	11	0.36	0.016
15–24	44	1.07	14	1.33	30	0.98	< 0.005
25–34	98	2.38	27	2.57	71	2.31	< 0.005
35–44	284	6.89	87	8.29	197	6.41	< 0.005
45–54	972	23.58	266	25.33	706	22.98	< 0.005
55–64	1302	31.59	345	32.86	957	31.15	< 0.005
65–74	917	22.25	209	19.90	708	23.05	< 0.005
75–84	451	10.94	92	8.76	359	11.69	< 0.005
85–98	39	0.95	6	0.57	33	1.07	< 0.005

*N* = 4122.

SD, standard deviation; DM, diabetes mellitus.

* , *p* < 0.05 was considered statistically significant.

† , Early onset DM includes all patients who were first diagnosed with diabetes when they were < 45 years old.

A total of 1514 (36.73%) African and 2493 (60.48%) Indian (South Asian) patients were included for further demographic stratification (Table 2). There was comparatively fewer mixed race and white (Caucasians) patients compared to African and Indian patients (South Asian), so they were excluded from further bivariate and multivariate analysis. Because of low numbers, mixed race and white patients were excluded from further analysis. Significantly, more Indian female patients were presented with T2DM compared with African female patients (*p* < 0.05). Similarly, more Indians were presented with T2DM in each age category compared to Africans. However, the early onset of T2DM was similar between African and Indian patients (214 and 215, respectively).

**TABLE 2:** Ethnicity-stratified demographic characterisation of all patients with type 2 diabetes mellitus.

Demographic	African		Indian		Mixed race		White	
	*n*	%	*n*	%	*n*	%	*n*	%
**Sex**								
Male	345	32.86	671	63.90	6	0.57	28	2.67
Female	1169	38.05	1822	59.31	18	0.59	63	2.05
**Early onset T2DM** †	214	14.13	213	8.54	2	8.33	12	13.19
**Age category (years)**								
0–15	9	60.00	6	40.00	0	-	0	-
16–24	31	70.45	12	27.27	1	2.27	0	-
25–34	46	46.94	49	50.00	0	-	3	3.06
35–44	128	45.07	146	51.41	1	0.35	9	3.17
45–54	373	38.37	584	60.08	4	0.41	11	1.13
55–64	541	41.55	721	55.38	11	0.84	29	2.23
65–74	267	29.12	617	67.28	5	0.55	28	3.05
75–84	109	24.17	330	73.17	2	0.44	10	2.22
85–98	10	25.64	28	71.79	0	-	1	2.56

*N* = 4122.

T2DM, type 2 diabetes mellitus.

† , Early onset DM includes all patients who were first diagnosed with diabetes when they were < 45 years old.

The categorised prevalence of comorbidities is presented in Table 3. Hypertension (3212) and cardiovascular problems (460) were most frequent, with a prevalence of 77.9% and 11.16%, respectively. Approximately 40% (176/441) of patients with early onset DM also suffered with hypertension. The prevalence of comorbidities, such as hypertension, arthritis and anaemia, was significantly higher amongst female patients than in male patients. Whilst cardiovascular problems were significantly higher for Indians compared to Africans (67% vs. 28%, *p* < 0.05), the converse was true for epilepsy and mental health. The likelihood of presenting with comorbidities increased significantly with age, as most patients aged between 55 and 74 years presented with hypertension, cardiovascular problems and arthritis (*p* < 0.05).

**TABLE 3:** Existing comorbidities amongst patients with type 2 diabetes mellitus.

Comorbidities	Hypertension		Cardiac		Epilepsy		Asthma		Arthritis		Anaemia		Mental health		TB	
	*n*	%	*n*	%	*n*	%	*n*	%	*n*	%	*n*	%	*n*	%	*n*	%
**Total**	3212	77.92	460	11.16	99	2.40	207	5.02	245	5.94	118	2.86	116	2.81	59	1.43
**Sex**																
Male	761	23.69	313	68.04	30	30.30	48	23.19	33	13.47	15	12.71	33	28.45	12	20.34
Female	2451	76.31	147	31.96	69	69.70	159	76.81	212	86.53	103	87.29	83	71.55	47	79.66
*p* *	< 0.005	-	< 0.001	-	0.264	-	0.439	-	< 0.005	-	< 0.001	-	0.456	-	0.362	-
**Race**																
African	1149	35.77	130	28.26	53	53.54	46	22.22	107	43.67	36	30.51	53	45.69	27	45.76
Indian	1976	61.52	309	67.17	43	43.43	151	72.95	134	54.69	82	69.49	55	47.41	31	52.54
*p* *	0.085	-	< 0.005	-	< 0.004	-	< 0.005	-	0.105	-	0.098	-	< 0.004	-	0.208	-
**Early onset DM**	176	40.00	21	4.76	8	1.81	24	5.44	9	2.04	10	2.27	8	1.81	3	0.68
**Age category (years)**																
0–15	1	0.03	1	0.22	-	-	-	-	-	-	-	-	-	-	-	-
15–24	9	0.28	1	0.22	-	-	-	-	-	-	-	-	3	2.59	-	-
25–34	30	0.93	2	0.43	-	-	3	1.45	-	-	3	2.54	1	0.86	-	-
35–44	136	4.23	17	3.70	8	8.08	21	10.14	9	3.67	7	5.93	4	3.45	3	5.08
45–54	710	22.10	90	19.57	26	26.26	53	25.60	55	22.45	30	25.42	30	25.86	17	28.81
55–64	1090	33.94	137	29.78	43	43.43	67	32.37	103	42.04	27	22.88	35	30.17	24	40.68
65–74	799	24.88	141	30.65	10	10.10	44	21.26	52	21.22	35	29.66	27	23.28	8	13.56
75–84	405	12.61	68	14.78	11	11.11	17	8.21	26	10.61	14	11.86	16	13.79	7	11.86
85–100	32	1.00	3	0.65	1	1.01	2	0.97	-	-	2	1.69	-	-	-	-
*p*	< 0.005	-	< 0.005	-	< 0.048	-	0.321	-	< 0.001	-	0.358	-	0.374	-	0.487	-

*N* = 4007.

DM, diabetes mellitus; TB, tuberculosis.

* , *p* < 0.05 was considered statistically significant.

When logistic regression was applied using sex and ethnicity (African and Indian) as independent variables, female patients with T2DM were at significantly higher risk of presenting with hypertension (odds ratio [OR] = 1.44, 95% CI:1.20;1.71), arthritis (OR = 2.20, 95% CI:1.51;3.20) and anaemia (OR = 2.42, 95% CI:1.40;4.19), whilst their risk for cardiovascular problems was significantly lower compared to male patients (OR = 0.67, 95% CI: 0.54;0.83). Age-adjusted regression also illustrated a higher risk for cardiovascular problems and asthma amongst Indians with T2DM, with a lower risk for epilepsy, arthritis, and mental health-related problems compared to Africans (Table 4). Table 5 illustrates the number of co-existing morbidities stratified by gender, ethnicity and age. Overall, only 621 (15%) patients had no comorbidity, whilst 2522 (61%) patients suffered at least one diagnosed condition. Hospital registers demonstrated more comorbidities for women compared to men (*p* < 0.05), with 84% (2574/3072) women presenting with T2DM and either 1 or 2 morbidities simultaneously. For both sexes, it was observed that DM plus one comorbidity was the most common combination. Indians presented with significantly more comorbidities than Africans. When the presence of multiple morbidities was further stratified by sex, significantly more Indian male patients presented with DM + 1 morbidity compared to African male patients (*p* < 0.005). Similar results with respect to ethnicity were obtained for all other categories of frequency of comorbidities when stratified by sex and age category (Table 6).

**TABLE 4:** Logistic regression analysis of comorbidities presented by patients with type 2 diabetes mellitus.

Existing comorbidity	Female		Indian		*p*
	OR	95% CI	OR	95% CI	
Hypertension	1.44	1.20;1.71	1.01	0.85;1.20	< 0.005
Cardiac	0.67	0.54;0.83	1.35	1.09;1.68	< 0.001
Epilepsy	0.76	0.48;1.18	0.48	0.32;0.73	0.264
Asthma	1.15	0.82;1.62	2.11	1.50;2.96	0.439
Arthritis	2.20	1.51;3.20	0.77	0.59;0.10	< 0.005
Anaemia	2.42	1.40;4.19	1.44	0.97;2.14	< 0.001
Mental health	0.82	0.54;1.25	0.62	0.42;0.90	0.456
TB	1.28	0.68;2.43	0.70	0.42;1.18	0.362

Note: Models were adjusted for age. Male patients and African patients were the reference categories.

*N* = 4022.

TB, tuberculosis; OR, odds ratio; 95% CI, 95% confidence interval.

* , *p* < 0.001;

** , *p* < 0.05.

**TABLE 5:** Type 2 diabetes mellitus patients stratified by frequency of comorbidities.

Demographics	Frequency of co-existing morbidities									
	DM only†		DM+1		DM+2		DM+3		DM+4	
	*n*	%	*n*	%	*n*	%	*n*	%	*n*	%
**Total**	621	15.07	2522	61.18	878	21.30	92	2.23	9	0.22
**Sex**										
Male	208	33.55	605	23.99	221	25.17	16	17.39	-	-
Female	412	66.45	1917	76.01	657	74.83	76	82.61	9	100.00
*p*	< 0.005	-	< 0.005	-	< 0.005	-	< 0.000	-	< 0.002	-
**Race**										
African	254	42.12	914	37.17	312	36.84	29	32.95	4	44.44
Indian	349	57.88	1545	62.83	535	63.16	59	67.05	5	55.56
*p*	< 0.005	-	< 0.005	-	< 0.005	-	< 0.001	-	0.739	-
**Early onset DM** ‡	227	36.55	164	6.50	44	5.01	5	5.43	1	1.11
**Age category (years)**										
0–15	13	2.10	2	0.08	-	-	-	-	-	-
15–24	32	5.16	11	0.44	-	-	1	1.09	-	-
25–34	62	10.00	30	1.19	4	0.46	1	1.09	-	-
35–44	119	19.19	121	4.80	40	4.56	3	3.26	1	11.11
45–54	170	27.42	597	23.67	187	21.30	15	16.30	3	33.33
55–64	125	20.16	845	33.51	291	33.14	39	42.39	2	22.22
65–74	67	10.81	592	23.47	240	27.33	15	16.30	3	33.33
75–84	28	4.52	294	11.66	112	12.76	17	18.48	-	-
85–98	4	0.65	30	1.19	4	0.46	1	1.09	-	-

*N* = 4022.

DM, diabetes mellitus.

* , *p* < 0.05 was considered statistically significant.

† , patients presenting with only DM; DM+1: patients presenting with DM and 1 comorbidity; DM+2: patients presenting with DM and 2 comorbidities; DM+3: patients presenting with DM and 3 comorbidities; DM+4: patients presenting with DM and 4 comorbidities

‡ , Early onset DM includes all patients under 45 years.

**TABLE 6:** Demographic profile of type 2 diabetes mellitus patients stratified by frequency of comorbidities.

Demographics	African								Indian							
	DM+1		DM+2		DM+3		DM+4		DM+1		DM+2		DM+3		DM+4	
	*n*	%	*n*	%	*n*	%	*n*	%	*n*	%	*n*	%	*n*	%	*n*	%
**Sex**																
Male	187	31.75*	69	32.39	6	40.00	1	-	402	68.25*	144	67.61	9	60.00	-	-
Female	727	38.88	243	38.33	23	31.51	4	44.44	1143	61.12	391	61.67	50	68.49	5	55.56
**Early onset DM** †	75	35.05	20	9.35	2	0.93	-	-	83	38.97	23	10.80	3	1.41	1	0.47
**Age category (years)**																
0–15	2	100.00	-	-	-	-	-	-	-	-	-	-	-	-	-	-
16–24	7	63.64	-	-	-	-	-	-	4	36.36	-	-	1	100.00	-	-
25–34	12	41.38	2	50.00	1	100.00	-	-	17	58.62	2	50.00	-	-	-	-
35–44	54	46.55	18	46.15	1	33.33	-	-	62	53.45	21	53.85	2	66.67	1	100.00
45–54	225	38.59	87	46.52	7	46.69	1	33.33	358	61.41	100	53.48	8	53.33	2	6.67
55–64	354	42.86	117	42.09	15	41.67	2	100.00	472	57.14	161	57.91	21	58.33	-	-
65–74	178	30.96	66	29.07	2	14.29	1	33.33	397	69.04	161	70.93	12	85.71	2	6.67
75–84	74	25.69	22	20.37	3	17.65	-	-	214	74.31	86	79.63	14	82.35	-	-
85–98	8	27.59	-	-	-	-	-	-	21	72.41	4	100.00	1	100.00	-	-

DM only: patients presenting with only DM; DM+1: patients presenting with DM and 1 comorbidity; DM+2: patients presenting with DM and 2 comorbidities; DM+3: patients presenting with DM and 3 comorbidities; DM+4: patients presenting with DM and 4 comorbidities.

*N* = 4122.

DM, diabetes mellitus.

* , *p* < 0.001;

** , *p* < 0.05;

*** , *p* < 0.005.

† , Early onset DM includes all patients under 45 years.

Age-adjusted regression analysis was done using sex and ethnicity as independent variables (Table 7). The logit estimate for female patients relative to male patients was significantly lower (β = ∡0.42, 95% CI: ∡0.62;–0.21) for presenting with DM only compared to DM + 1 comorbidity (*p* < 0.001), which indicates that male patients are more likely than female patients to present with DM only. None of the other regression analyses using sex and ethnicity were statistically significant.

**TABLE 7:** Regression analysis of the number of comorbidities (frequency) stratified by sex and ethnicity.

Frequency of comorbidities†	Female		Indian	
	β-coeff	95% CI	β-coeff	95% CI
DM only‡	−0.42*	−0.62;–0.21	0.06	−0.13;0.25
DM+2	−0.07	−0.25;0.10	−0.01	−0.18;0.15
DM+3	0.4	−0.15;0.95	−0.2	−0.28;0.63

Note: Reference categories were male patients and African patients.

DM, diabetes mellitus; 95% CI, 95% confidence interval.

* , *p* < 0.001.

† , The frequency of DM + 1 comorbidity was the base outcome. DM+4 was excluded because of low frequency.

‡ , Patients presenting with only DM; DM+1: patients presenting with DM and 1 comorbidity; DM+2: patients presenting with DM and 2 comorbidities; DM+3: patients presenting with DM and 3 comorbidities; DM+4: patients presenting with DM and 4 comorbidities.

## Discussion

This study demonstrates the demographically stratified prevalence of T2DM and existing comorbidities amongst an outpatient population in a district healthcare facility in KZN. Our data highlight that more women than men present with T2DM and one or more of the existing comorbidities. Even though all reported existing morbidities are not directly linked with T2DM, it impacts on healthcare resources, particularly for treatment modalities. Moreover, this was shown to be significantly influenced by demographic factors such as sex, ethnicity and age. Indians were overrepresented in the population under study, and logistic regression showed a greater risk of disease burden amongst Indians compared to Africans, particularly with respect to hypertension. The high burden of T2DM and hypertension as coexisting morbidities (77.9%) and the 10% prevalence of early onset DM (patients < 45 years) should be noted as serious concerns in an epidemiological transition in SA where non-communicable diseases (NCDs) are increasingly prevalent.

T2DM prevalence has been reported in several studies conducted in South Africa;^24,29^ however, incidence data have been limited. Despite the recent reporting incidence of DM for 11 districts in KZN by Sahadew et al.,^5^ the data are limited in which only DHIS data were used. District Health Information Systems data contain patient visits for T2DM treatment aggregated per healthcare facility and exclude individual patient tracking through identifiers. Thus, it may be possible that a single patient could be counted several times as patients may report to the facility on a monthly basis to collect their medication. This study presents T2DM patients who were given unique hospital identifiers at their first visit for treatment. Moreover, we chose a 6-month period as some diabetics would only see a doctor once every 6 months, whilst continuing to collect their chronic medication at an affiliated clinic.

Our data revealed a significantly higher prevalence of T2DM amongst female patients in contrast to male patients, irrespective of age and sex. This may be associated with sociocultural factors such as varying behavioural patterns between male patients and female patients which influences their nutritional patterns, lifestyle and attitudes towards treatment and prevention.^30^ Access to healthcare differs amongst male patients and female patients, as a result of their personal preconceptions.^31^ Hi-tech and rapid remedies are usually pursued by men in contrast to the extensive sociocultural therapies pursued by female patients.^31^ It is believed that the sociocultural female nature permits an escape from financial and societal drawbacks, whereas the masculine nature of males prompts the search for care that warrants a comprehensive speedy recuperation and lower economic burden.^31^ In addition, health-seeking behaviour may be higher for women compared to men in South Africa, particularly with respect to chronic diseases.^32^ Hence, it is possible that sociocultural and socioeconomic factors combined with spiritual beliefs and semantics are instrumental in understanding the bolder health-seeking behaviour represented by the higher percentage of women in our sample.

African data for gender distribution in T2DM have reported conflicting results. An increased prevalence amongst male patients was reported from studies in Tunisia, Egypt, Sudan, Cameroon and rural Tanzania,^14,33,34,35^ whilst prevalence was greater amongst female patients in South Africa (Durban), Mali, Cameroon and Sudan^14,34,36^ with an equal gender distribution reported from South Africa (Cape Town), Tanzania and Sudan.^15,30,36^

Recent reports suggest that diabetics are predisposed to one or more comorbidity.^37^ A study conducted in 12 primary healthcare clinics in South Africa revealed a 79% prevalence of hypertension, whilst complications related to the eyes, feet and kidneys were 8.2%, 6.5% and 21.4% respectively.^34^ Data on comorbidities-linked to eyes, feet and kidneys were not available for this study as we were limited by the hospital-outpatient register. T2DM is reported as the seventh leading cause for the risk of increased infections, morbidity and mortality in South Africa^24^ and accounted for approximately 68 000 deaths in 2013.^5^ However, this may be a significant underestimation, when considering the mortality from complications or comorbidity associated with DM. The high prevalence of hypertension (77.9%) amongst patients should be highlighted, particularly that 80% women (2451/3072) and 40% of all early onset diabetics (176/441) presented with hypertension. Our data are in contrast with earlier data from ref. 38, which suggested that hypertension affects 20%–60% of patients with T2DM, depending on obesity, ethnicity and age. It would appear that morbidity associated with hypertension, which is a common complication of T2DM, is increasing. This is of concern as the combination of DM and hypertension increases the risk of premature cardiovascular disease.^38^

Regression analyses demonstrated that female patients with T2DM were at significantly higher risk of presenting with hypertension (OR = 1.44, 95% CI:1.20–1.71, *p* < 0.001) and arthritis but had a lower risk for cardiovascular problems compared to male patients (OR = 0.67, 95% CI: 0.54–0.83, *p* < 0.001). Likewise, the risk of cardiovascular disease (CVD), especially myocardial infarction, was also shown to be greater in a Danish cohort of female diabetic patients below 50 years.^39^ More recently, female patients already diagnosed with T2DM develop a greater risk to acquire CVD, with overweight/obesity and postmenopausal women being at a higher risk.^40^ We also found that the prevalence of comorbidities was highest amongst Indians regardless of age and sex, in contrast to other race groups. Regression data further demonstrated that Indians with T2DM were predisposed to a higher risk for cardiovascular problems and asthma, but had a lower risk for epilepsy, arthritis and mental health issues compared to Africans. The 12.5% crude prevalence of T2DM in KZN was higher than the 9.2% national prevalence, which could be because of the large population of Indians (South Asian) living in KZN in contrast to other cities in SA, suggestive of a possible genetic predisposition to T2DM.^5,15^ Our data suggest that comorbidities increase significantly with age, which is in agreement with the study made by Uddin et al.^41^ Uddin and co-workers reported that T2DM + 1 and T2DM + 2 comorbidities were higher in male patients, whilst DM + 3 comorbidities were higher amongst female patients.^41^ However, our data showed significantly more female patients with T2DM + 1, T2DM + 2, T2DM + 3 comorbidities (*p* < 0.05), which was suggestive that sex, ethnicity and age are critical determinants associated with the prevalence of T2DM and comorbidities.

Demographic data are essential in advancing the precision in the prediction of incidence, complications, morbidity and mortality related to T2DM.^20^ Data obtained from such analyses will accurately inform healthcare systems and enhance the development of tracking proficiencies required for reducing the incidence of DM and associated comorbidities. To date, the management of T2DM following a uniform treatment algorithm is usually associated with poor treatment adherence and the subsequent development of complications.^42^ Recent data suggest that some medications may serve dual purposes, for example, the use of metformin (a first line treatment for T2DM) reduced asthma-related outcomes in patients who presented with T2DM and asthma concurrently,^43^ whilst hydroxychloroquine (commonly used antirheumatic medication) demonstrated hypoglycaemic effects.^44^ The healthcare system in SA, which is already burdened by the HIV/TB epidemic coupled with increasing infectious diseases, could be further constrained by the increasing prevalence of diabetes and comorbidities. Various studies conducted in South Africa, Nigeria, Ghana, Cameroon and Tanzania confirm both the increase in prevalence and the changing epidemiology of diabetes complications;,^17^ however, these data are over 15 years old. Arising from these predictions, it is crucial to re-evaluate the extent of the problem so that healthcare resources may be appropriately allocated. In order to gain better control of chronic complications, treatment and management for T2DM and complications, interventions should primarily target the highly prevalent populations with chronic complications including older diabetic patients and those with a long history of diabetes.

A limitation of this study is that as this study was hospital-based, the results only apply for diabetics requiring primary level of healthcare rather than representing the total population of diabetics which include people able to access medical aid and private healthcare. In addition, hospital outpatient records did not include information about microvascular conditions that are a significant comorbidity related to diabetes-linked mortality and morbidity.

## Conclusion

The variation of complications with age, gender and ethnicity amongst patients with a T2DM diagnosis all point to a need for flexible and adaptive approaches for the prevention and management of T2DM cases in order to allocate medical resources efficiently and according to the true local burden of disease because of T2DM complications. Findings arising from this study are specific to the Indian and African populations in KZN, but may be aligned with some from other provinces in South Africa. Future research evaluating long-term clinical outcomes in high-risk sub-populations, based on ethnicity, age and underlying comorbid conditions, is warranted for more effective management. Furthermore, the disparities in comorbidities linked with T2DM based on ethnicity warrant the exploration of precision medicine amongst indigenous populations in South Africa. The use of precision medicine in stratifying diabetics into groups based on the molecular and genetic biomarkers as well as clinical characteristics has been explored to optimise therapeutic outcomes, with the intention of providing recommendations that target groups rather than individual patients. This may be an exploratory option for SA where the increasing prevalence of T2DM, coupled with the attributable burden of associated comorbidity, has the potential to create significant pressure on the healthcare system.
